# Dynamic Effects of CYP2D6 Genetic Variants in a Set of Poor Metaboliser Patients with Infiltrating Ductal Cancer Under Treatment with Tamoxifen

**DOI:** 10.1038/s41598-018-38340-6

**Published:** 2019-02-21

**Authors:** Yeimy Viviana Ariza Márquez, Ignacio Briceño, Fabio Aristizábal, Luis Fernando Niño, Juvenal Yosa Reyes

**Affiliations:** 1 Universidad Nacional de Colombia, Instituto de Biotecnología IBUN, Departamento de Farmacia, Bogota, 111321 Colombia; 2 Universidad de la Sabana, Facultad de Medicina, Bogota, 140013 Colombia; 3 Pontificia Universidad Javeriana, Facultad de Medicina, Instituto de Genética Humana IGH, Bogota, 110231 Colombia; 4 Universidad Nacional de Colombia, Facultad de Ingeniería, Departamento de Ingeniería de Sistemas e Industrial, Bogota, 111321 Colombia; 5 Universidad Simón Bolivar, Facultad de Ciencias Básicas y Biomédicas, Laboratorio de Simulación Molecular y Bioinformática, Barranquilla, 080002 Colombia

## Abstract

Breast cancer is a group of multigenic diseases. It is the most common cancer diagnosed among women worldwide and is often treated with tamoxifen. Tamoxifen is catalysed by cytochrome P450 2D6 (CYP2D6), and inter-individual variations in the enzyme due to single nucleotide polymorphisms (SNPs) could alter enzyme activity. We evaluated SNPs in patients from Colombia in South America who were receiving tamoxifen treatment for breast cancer. Allelic diversity in the CYP2D6 gene was found in the studied population, with two patients displaying the poor-metaboliser phenotype. Molecular dynamics and trajectory analyses were performed for CYP2D6 from these two patients, comparing it with the common allelic form (CYP2D6*1). Although we found no significant structural change in the protein, its dynamics differ significantly from those of CYP2D6*1, the effect of such differential dynamics resulting in an inefficient enzyme with serious implications for tamoxifen-treated patients, increasing the risk of disease relapse and ineffective treatment.

## Introduction

Breast cancer (BC) is a group of multigenic diseases, with a significant public health impact worldwide and the highest cancer-related mortality in women in South America^1^. BC depends on oestrogen and progesterone for their growth and replication^2^. Nearly 75% of all breast cancers are oestrogen receptor positive (ER+), 65% of which are also progesterone receptor positive^3^. Approximately 1.67 million new cancer cases are reported each year, 9.1% of which occur in Latin America and the Caribbean^4^. BC is the most common cancer among women and the leading cause of cancer-related death worldwide^1^. Central and South American countries, especially Colombia, have experienced an epidemiological and demographic transition, likely due to rapid economic growth, associated with a high number of BC cases^5–7^. In 2012, BC was the most common cancer diagnosed and the leading cause of cancer-related deaths in females, with over 140,000 new breast cancer cases and nearly 40,000 related deaths occurring in Central and South America^1^. Reports published in 2012 indicate that the mortality rates for BC are increasing^8–12^. By 2030, the new cases of female BC in Central and South America are estimated to increase by 70% (224,000 new cases and 66,000 deaths) due to demographic changes^1^.

Hormone therapy is widely used to treat BC, and tamoxifen is currently used for treating both early and advanced ER+ BC in pre- and post-menopausal women^13^. Tamoxifen therapy reportedly decreases the risk of disease relapse, some research suggest that tamoxifen therapy decreases the risk of BC relapse, in which it has been reported a decrease of 2% in the annual rate of distant relapse in premenopausal women (ER+ invasive tumors) after 5 years of treatment with tamoxifen, particularly for invasive ER+ tumours in premenopausal women^14,15^. Tamoxifen is metabolised into the active metabolites hydroxytamoxifen and endoxifen, which have 100- fold greater affinity for the ER and 30- to 100-fold greater potency for inhibiting oestrogen-dependent cell growth than the precursor tamoxifen. Tamoxifen is metabolised by the cytochrome P450 isoforms CYP2D6 and CYP3A4^16^. The underlying mechanisms of tamoxifen resistance include tumour- and host genome-associated factors^17^. Pharmacogenetics research on the relationship between CYP2D6 polymorphisms and the efficacy of tamoxifen in early BC has shown a strong relationship between a patient’s capacity to metabolise tamoxifen and treatment outcome^18^. To date, over 140 allelic variants of CYP2D6 have been described, and several of them are associated with reduced or no activity. These complex pharmacogenetic relationships have been established by the efficiency of tamoxifen transformation, which can be correlated with CYP2D6 genes and single nucleotide polymorphisms (SNPs) association^17–21^.

Several Molecular Dynamics MD simulation studies have been carried out on CYP proteins with and without SNPs. The simulation results, pointed out the importance of the structure and motion for catalytic action in the protein^22^. Also, according to MD simulations and experimental data, it is suggesting that changes in CYP proteins, is due to the ability of the enzyme to simultaneously bind two substrate molecules. Thus, SNPs can affect such bound due to change in the dynamics^23,24^. For example, for YP3A4, CYP2C9, CYP2A6 and CYP2D6, compared with the prevalent allelic form 1*, flexibility due to SNPs is affected and possibly disrupting the ligand entrance to the active site^25–27^.

Here, we investigated patients based on CYP2D6 metaboliser phenotypes^19^, and the relationship with the plasticity and tamoxifen entrance affectation to the catalytic pocket. Patients with an extensive metaboliser (EM) phenotype carry two functional CYP2D6 alleles (CYP2D6 *1, *2, *33 or *35); those with an intermediate metaboliser (IM) phenotype carry reduced function alleles (CYP2D6 *9, *10, *17 or *41) and those with a poor metaboliser (PM) phenotype carry two or one deficient allele (CYP2D6 *3, *4, or *5) and at least one reduced function allele; this latter group may benefit less from tamoxifen. Importantly, pharmacokinetics studies have revealed that CYP2D6 genotypes are associated with different concentrations of tamoxifen metabolites, mainly endoxifen, in a patient-dependent manner^2,18,20,21^. A subset of the most common alleles was selected for phenotype prediction and was compared with information reported in other human populations (see Supplementary T1). Alleles were grouped according to their perceived functionality, wherein PM included at least one allele with null function. These terms were determined by the Clinical Pharmacogenetics Implementation Consortium (CPIC) in an effort to standardise terms for reporting clinical pharmacogenetic test results^28^. The frequency of the CYP2D6 variants in different populations has been described in previous studies; the CYP2D6*4 allele was most common in Caucasians, CYP2D6*10 in East Asians, CYP2D6*41 and duplication/multiplication of active alleles in Middle Easterners, CYP2D6*17 in Black Africans and CYP2D6*29 in African Americans. Overall, the PM phenotype is more frequent among Caucasians, and the ultra-fast metaboliser (UM) phenotype is more frequent among Middle Easterners and Ethiopians^19^.

In Colombia, one study evaluated CYP2D6 in 121 healthy individuals from the city of Pereira and identified CYP2D6 *1, *2, *3, *4, *5 and *17 alleles^29^. Similarly, two studies that attempted to characterise the Embera and Ngawbe communities identified the variants CYP2D6 *4, *6 and *10^30^. Finally, studies involving 123 participants from the academic and student staff at Pontificia Universidad Javeriana (Bogota) and 148 participants of the Colombian Air Force school identified the CYP2D6 *2, *3, *4, *5, *6, *10, *35 and *41 alleles^31,32^. Despite these studies, there is a lack of information about CYPD26 variants in Colombian population with BC and the effectiveness of tamoxifen. Here, we studied 30 patients at the San Ignacio University Hospital who were diagnosed with infiltrating ductal cancer and treated with tamoxifen. The goal was to identify the genotypes and intermediary metabolites of tamoxifen in this Colombian population and to define the influence of CYP2D6 mutations on the enzyme’s structure and dynamics.

## Results

### Patient characteristics

The clinical characteristics of the 30 patients with infiltrating ductal carcinoma are summarised in Table 1. The mean age of the patients was 60.9 (range: 42–81) years. At the time of blood draw, the mean body mass index (BMI) was approximately 26.33 kg/m^2^ (16.22–38.13 kg/m^2^), and the mean duration of tamoxifen treatment was 16 months (range, 4–60 months). Most patients were menopausal (93.3%), no patients reported taking CYP2D6 inhibitors, and 14 patients used concomitant medication with tamoxifen.

**Table 1:** Clinical characteristics of the 30 patients with breast cancer included in the study.

Characteristics	Mean (range) or n (%)
BMI (kg/m2)	26, 33 (16, 22–38, 13)
Age (Years)	60, 9 (42–81)
Menopausal status, n (%)	
Pre-menopause	2, (6.3)
Post-Menopause	28 (93.7)
Concomitant medicine, n (%)a	
0 = (no)	16 (53.3)
1 = (yes)	14 (46.7)
CYP inhibitor	0 (0)
Tumor size	
<2 cm	22 (73.3)
>2 cm >5 cm	8 (26.7)
Lymph nodes	
0	26 (86.7)
1	4 (13.3)
Stage	
I	13 (43.3)
II	13 (43.3)
III	4 (13.4)

### Plasma concentrations of intermediary metabolites

A total of 30 evaluations of tamoxifen and its metabolites were performed. All patients were previously dosed with 20-mg tamoxifen daily for at least 4 months (Table 2). Among the three tamoxifen metabolites, N-desmethyl-tamoxifen had the highest mean concentration (45.26 ng/mL) compared with endoxifen (3.17 ng/mL) and 4-hydroxy-tamoxifen (1.2 ng/mL). Eight patients (patients 3, 4, 15, 17, 18, 20, 26 and 29) with metabolite levels <10 ng/mL were not included in the analysis. The concentrations of intermediary metabolites of endoxifen are reported in Fig. S1 (see Supplementary S1). A comparison of the three metabolising states [0, PM (Poor); 1, IM (Intermediate); and 2, EM (Extensive)] showed that endoxifen concentration increased in the EM and IM patients, indicating that tamoxifen had been metabolised. However, the tamoxifen concentration varied, and this could be due to differences in each patient’s adherence to drug use. Consequently, patients who presented metabolite levels <10 ng/mL were not included in the analysis.

**Table 2:** The distributions of concentrations of metabolites of tamoxifen in 30 plasm specimen of 30 patients. NDM: N-desmethyltamoxifen; Tam: tamoxifen, 4 OH Tam: 4-hydroxytamoxifen; SD: standard deviation; CV: coefficient of variation.

Characteristics	Concentration (ng/mL)			
	Endoxifen	NDM	TAM	4-OH-TAM
Mean	3.17	45.26	31.47	1.12
SD	2.48	35.48	26.24	0.43
CV	78%	78%	83%	38%

### Genotype frequencies

From blood samples, we identified CYP2D6 variants with normal function. Table 3 (genotyping information is shown in T2 Supplementary). Specifically, CYP2D6*1 had a frequency of 0.393, CYP2D6*2A of 0.295 and CYP2D6*35 of 0.032. Likewise, we identified non-functional variants; CYP2D6*3 had a frequency of 0.032 and CYP2D6*4 of 0.016. Finally, we identified variants with reduced activity: CYP2D6*9 (0.032), CYP2D6*17 (0.016) and CYP2D6*41 (0.06). We could not identify the genotype for one patient, and one other sample could not be amplified. Importantly, this is the first time that the CYP2D6*9 variant has been identified in a Colombian population. Here, we used the following metabolising states: 3 = UM, presence of more than two alleles; 2 = EM or normal metaboliser, two alleles with normal function; 1 = IM, a combination of one allele with normal function and one with decreased or no activity; and 0 = PM, one allele with decreased activity and one non-functional allele or two non-functional alleles. We found that 17 of our patients were IMs, eight were EMs, two were PMs and one was UM; two were not assigned any genotype, association between metabolite concentration and metaboliser status for extensive and poor metabolisers is found in Fig. S1 (see Supplementary Fig. S1).

**Table 3:** Genotype frequencies of the CYP2D6 gene. UM: ultrafast metaboliser, EM: extensive metaboliser, IM: Intermediate metabolisers, PM: poor metaboliser, NI: Non- identified, NA: Non-amplified.

	Genotype	Predicted phenotype	n	%
CYP2D6	*1/*1	EM/EM	4	13.2
	*1/*2A	EM/EM	5	16.6
	*2A/*2A	EM/EM	4	13.2
	*1/*35A	EM/EM	1	3.3
	*2A/*35A	EM/EM	1	3.3
	*2A/*2A/2A	UM	1	3.3
	*1/*4	EM/PM	4	13.2
	*2A/*17	EM/IM	1	3.3
	*2A/*4	EM/PM	1	3.3
	*4/*9	PM/IM	1	3.3
	*1/*9	EM/IM	2	6.6
	*1/*3	EM/PM	2	6.6
	*4/*41	PM/IM	1	3.3
	NI		1	3.3
	NA		1	3.3

We found that in the PM group, the average endoxifen concentration was approximately 2.57 ng/mL, similar to that reported in previous studies. For example, Antures *et al*. found an endoxifen concentration of approximately 2.25 ng/mL in a Brazilian population, and Henning *et al*. reported an endoxifen concentration of 2.3 ng/mL in a group of Polish women^33^. Similarly, a study performed in the Netherlands and Belgium by Dezentje *et al*. showed that an average endoxifen concentration of approximately 2.5 ng/mL in women^21,34–36^. In contrast, studies performed by Lyon *et al*. showed a higher endoxifen concentration for different metabolisers.

**Figure 1:**
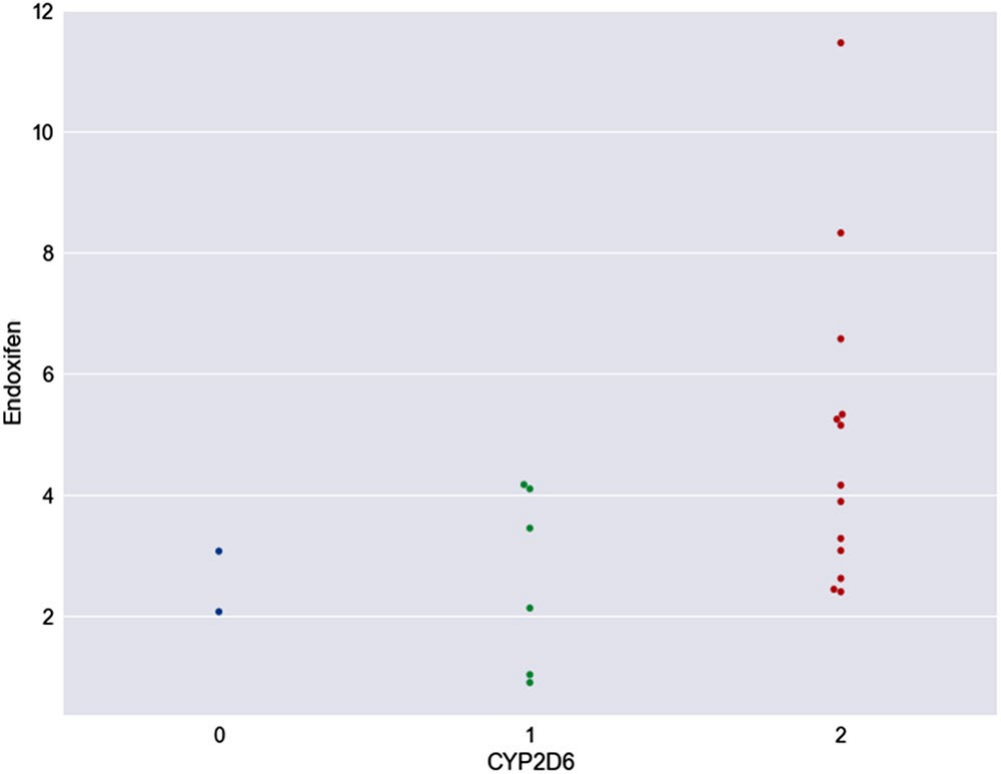
Scatter plot shows in the Y axis endoxifen concentration (*μ*g/ml), for each metabolising group in the phenotypes on the X axis of CYP2D6, where 2 = extensive metaboliser, 1 = intermediate metaboliser and 0 = poor metaboliser.

It is important to assign phenotypic classifications in relation to the genotype status to establish the similarities of the contrasted populations. In one study involving the Brazilian population, for example, the *4/*41 genotype was assigned the IM phenotype^37^. However, here we considered these patients to be PMs. Consequently, endoxifen concentrations also differed, which may indicate that the genotype affects the endoxifen concentration. Nonetheless, the endoxifen concentration in samples from IMs showed a mean deviation towards lower values (1.94 ng/mL) than expected (4.43 ng/mL). Here, the limitation of the data should be taken into account as variable adherence to the drug may affect the plasma endoxifen concentrations. After conducting telephone interviews with patients who had low concentrations of intermediaries metabolites we found out that they did not consume the medication regularly. Also, it should be noticed that in previous studies where the measurement of endoxifen showed higher concentrations, in the presence of more favorable phenotypes when the population was stratified by race/ethnicity, the Hispanic group showed low concentrations. Therefore, metaboliser phenotype alone may not be sufficient to determine whether tamoxifen is of potential benefit to an individual patient^21^. In addition, possible concomitant consumption of other drugs metabolised by CYP2D6 should be taken into consideration^38,39^. In addition, samples were collected from patients at different times of the day, adding further variability to the concentrations observed in the population^40,41^. In addition to classifying patients by metaboliser status, we also classified them by endoxifen level (Fig. 1). Eight patients with a tamoxifen concentration <10 ng/mL were excluded from this analysis due to the possibility that they were not taking the medication as prescribed.

Based on endoxifen levels, the following classifications were made (Fig. 1). Two patients with the lowest endoxifen concentrations were classified as PMs (0), six with intermediate endoxifen concentrations were classified as IMs (1) and 13 with the highest endoxifen concentrations were classified as EMs (2). One patient was not classified because genotyping was not possible.

Next, we investigated CYP2D6 SNPs and deletion in the two PM patients to better understand the effects of allelic variants on the biological activity of CYP2D6. Using molecular simulations, we identified *in silico* how SNP-induced changes affect the normal activity of the enzyme. The SNPs in patient 1 were Ser-488-Thr(rs1135840)/Arg-329-Cys(rs16947)/Pro-487-Ser(rs1065852) (termed Mut1)^42–44^, and those in patient 2 with one deletion were Ser-488-Thr(rs1135840)/Glu-253-Lys(rs3892097)/Pro-487-Ser(rs1065852)/Deletion-Leu-241(hCV32407229-rs5030656) (termed Mut2)^42,44–46^, (see Supplementary Fig. S2), those SNPs and deletion are located distal from the active site. We refer to the regular allelic form as 1*.

### Computational modeling

**Figure 2:**
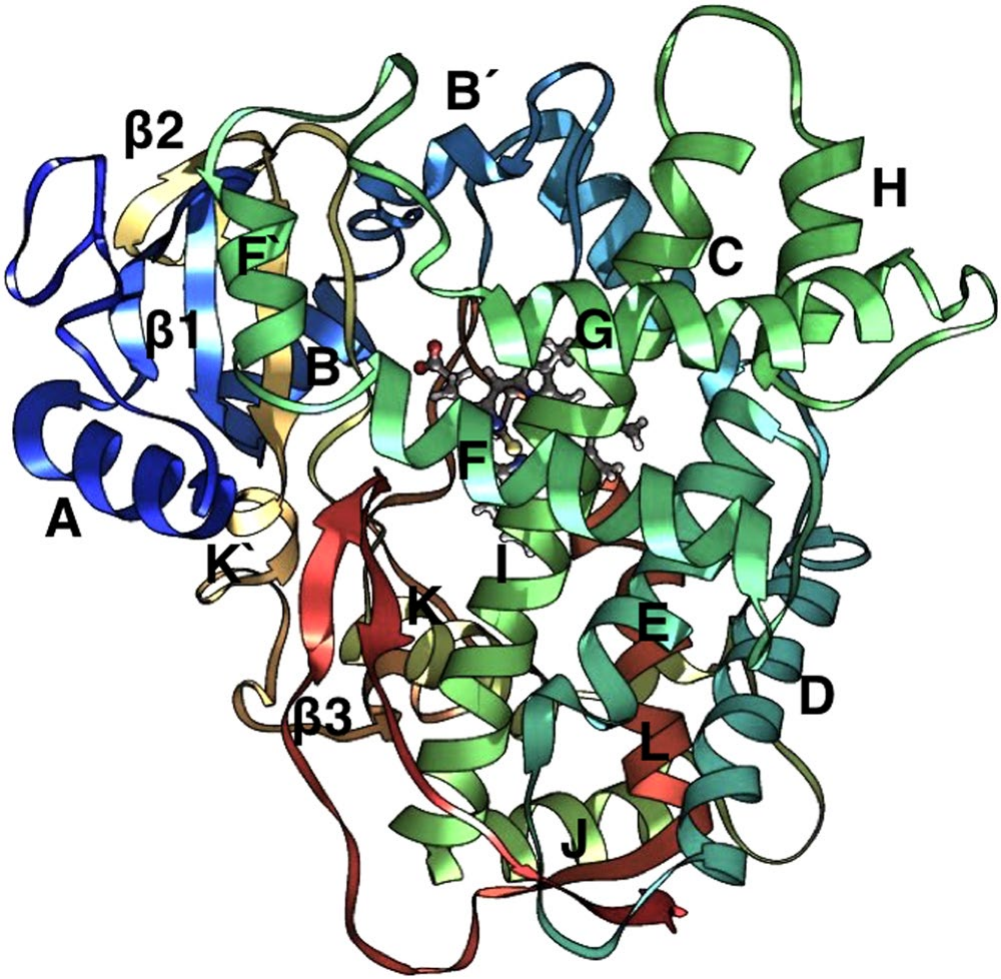
Topology is illustrated by the structure of Human Cytochrome P450 (CYP) 2D6-Prinomastat Complex (Protein Data Bank code 3QM4). Nomenclature was adopted from^47^.

The structure of 1* is shown in Fig. 2. The overall topology is illustrated, and the secondary structural components are similar to those of other cytochrome p450s^47^. The root mean square deviations (RMSDs) for 1*, Mut1 and Mut2 are shown in Fig. S3 (see Supplementary Fig. S3). As the protein model was built from the prinomastat bound crystal structure (3QM4), some differences were found when the average structure of the whole trajectory was compared with the crystal and MD simulations carried out by other authors. First, the 1* model was superimposed on the 3QM4 structure excluding the prinomastat ligand (see Supplementary Fig. S4). A comparison between the two structures is in agreement between the crystal structure and model previously described^25,27^. The RSMD value was about 1.113 Å, similar to the value reported by De Waal *et al*.^27^. Overall the structure of the 1* folds the same as 3QM4. Small differences were found concerning the structure of the 1* compared with the crystal, which notably is related and was reported by previous studies^25,27,48^. Helix F′ for example, in the *1 model was displaced 2.2 Å, value that was described by De Waal *et al*. as 3.5 Å and appear to be connected with F-G loop region, indicating that this part of the protein is flexible as we demonstrate later (see discussion). Helix G″ was shorter in the *1 model and displaced compared with 3QM4 crystal structure, indicating that this part of the protein is flexible too. The same results were described by other authors previously, which is a great validation, indicating that simulation performed here, described correctly the 1* allelic form^25,27,48^.

**Figure 3:**
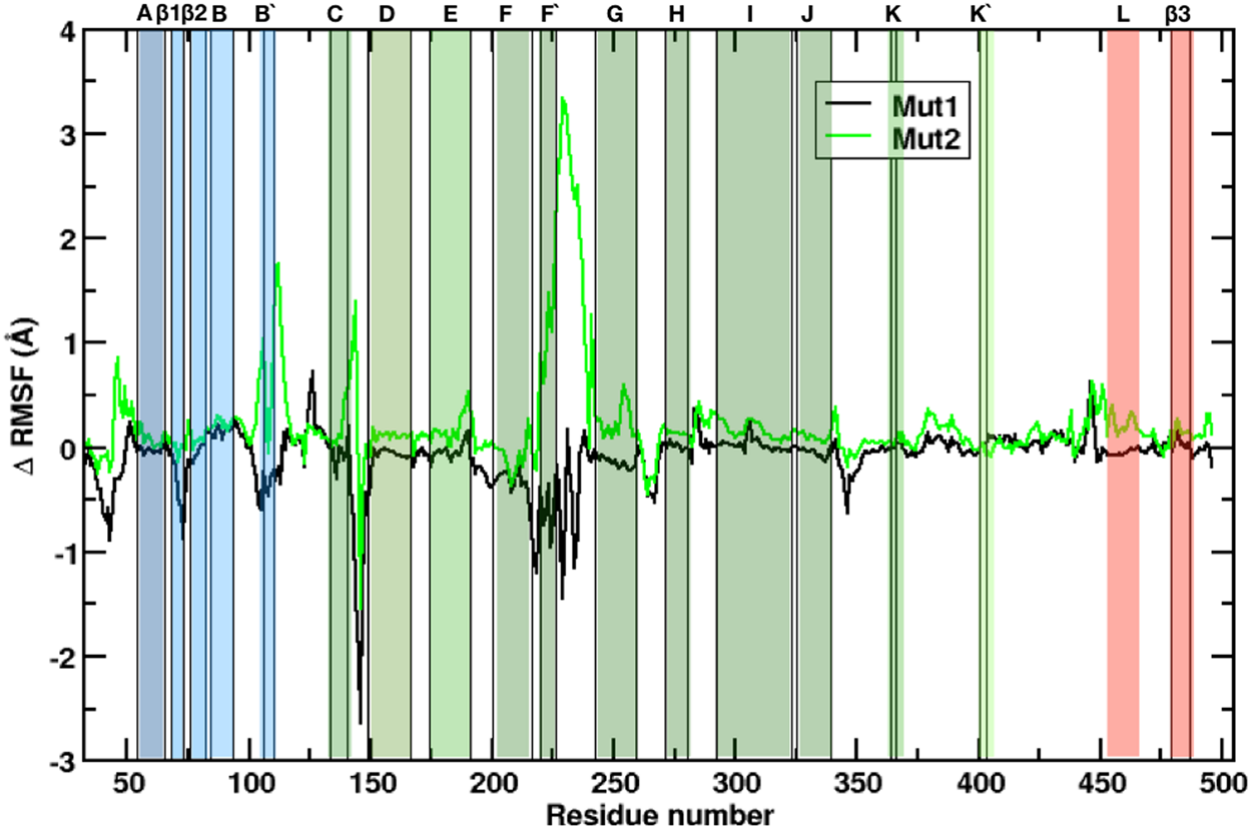
Changes in root mean square fluctuations (ΔRMSF) of backbone atoms in Mut1 (Black), Mut2 (Green), Color scheme follows the secondary structure in Fig. 2. Positive values of ΔRMSF correspond to the flexible zone as compared with 1*, while negative values of ΔRMSF corresponds to a rigid zone, also when compared to 1*.

All three structures equilibrated at 15, 5 and 42 ns for the allelic forms 1*, Mut1 and Mut 2, respectively. Data analysis of the trajectories was performed starting from 50 ns for the three simulations. The root mean square fluctuation (RMSF) is shown in Fig. S5 (see Supplementary Fig. S5) for the three allelic forms, and the RMSF difference ΔRMSF is shown in Fig. 3. The RMSF was computed using the value per residue of the allelic form 1* as a reference. Changes in plasticity were observed for Mut1 and Mut2 compared with 1*. For Mut1, rigid sites were observed, specifically in *β*1 (residues 36–48), helix B′ (residues 105–110), B′-C loop (residues 111–114), C-D loop (residues 142–150), F-F′ loop (residues 217–219), helix F′ (residues 220–226) and the F′-G loop (residues 227–241) (Figs 3 and S6 (see Supplementary Fig. S6)). Fig. S6 was constructed by populating the B-Factor column on the pdb files for the three allelic forms. In the case of Mut2, compared with Mut1, highly flexible sites were observed in the protein; specifically in *β*1 (residues 36–48), helix B′ (residues 105–110), B′-C loop (residues 111–114), C-D loop (residues 142–150), F-F′ loop (residues 217–219) and helix F′ (residues 220–226) (Figs 3 and S6B (see Supplementary Fig. S6)). Interestingly, there is no significant change in the catalytic pocket, compared with 1* and the crystal 3QM4 (video 1 and Fig. S6 (see Supplementary Fig. S6 and catalyticSite), which means that the distal SNPs and deletion for Mut2, does not have any effect directly in the catalytic site.

The largest negative value for the ΔRMSF was approximately −2.6 Å and was found in the C-D loop of Mut1. In contrast, the largest positive ΔRMSF, approximately 3.5 Å, was found in the F-F′ loop and helix F′ of Mut2. The negative values of ΔRMSF represent rigid dynamics behaviour, compared with the reference 1*, where the highest amount of flexibility is located above the cytochrome molecule and where ligand entrance tunnels are found^47^. Similar to the behaviour of Mut1, Mut2 shows greater flexibility, almost at the same sites as those in Mut1. Fig. S6C (see Fig. S6) shows a dramatic change in plasticity in which the B′ helix, F′-G loop and F′ helix are the largest flexible sites compared with the reference 1*.

**Figure 4:**
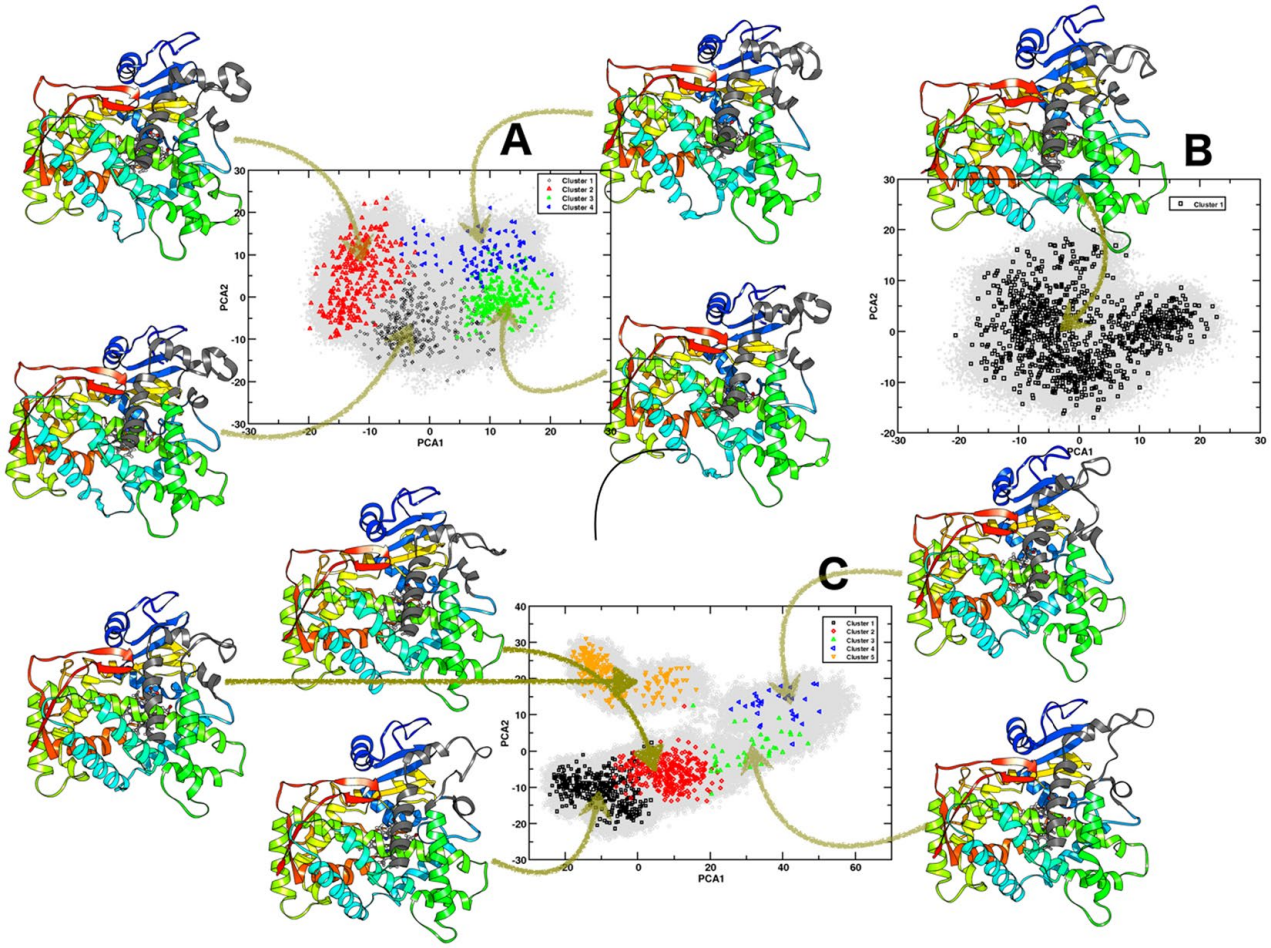
Projections of molecular dynamics trajectories and centroids structures onto the principal planes defined by the two most significant principal components, for (**A**) 1*, (**B**) Mut1 and, (**C**) Mut2. Projection of different clusters determined by k-means are shown in schematic colors depending on the number of clusters that were found for each trajectory; the projection was done for the subset of 1000 structures. The representative structure for each centroid cluster is also outlined, the principal structure differences observed in each centroid is shown in dim-gray for each structure.

Figure 4 shows the projections of molecular dynamics trajectories and centroid structures onto the principal planes defined by the two most significant principal components generated for the allelic form 1* (Fig. 4A), Mut1 (Fig. 4B) and Mut2 (Fig. 4C). Large differences were evident, especially for Mut2 with respect to 1*, as suggested from the plotted data along the direction of the two principal components. The eigenvector variations indicate the differences in motion between the 1*, Mut1 and Mut2 structures (Fig. 4). Low frequencies are sufficient to capture the principal protein motions along specific directions, which are represented by eigenvectors, as shown in the porcupine plots for the first three low frequency modes (see Supplementary Fig. S7). The eigenvectors show a difference in the direction of motion for Mut1 and Mut2 compared with 1*, which is consistent with the principal component analysis (PCA) scatter plot.

Cluster analysis (using k-means) was performed for a subset (1000 structures), which represented a sample of the complete data set, with a fixed cluster radius of 1.7 Å; this analysis was performed for the three allelic forms (Fig. 4). For the allelic form 1*, four clusters were identified and projected onto the PCA plot of the complete data set. The common four cluster structures are represented with the centroid extracted from each cluster (Fig. 4A). The four centroids plots show differences in the position of the F-F′ loop, F′ helix, B′ helix, F′-G loop and a deformation of helix F. Previous studies have shown that the F-G loop acts as a ‘hatch’ for ligands to enter at the enzyme active site^22^, which accounts for the large motion observed from one cluster to the other, as it is also observed in the RMSF and porcupine plots (see Supplementary Figs S6 and S7). The deformation of the F helix were found close to the solvent tunnel described by Johnson and Stout^47^, which is probably related to the opened and closed forms of the ligand entrance tunnel. Video 2 shows the motion for the 1,000 frames (see Supplementary videoMovieWild).

In the case of Mut1, a single cluster was found, suggesting a restrictive motion compared with 1*. The movements of the F-F′ loop, F′ helix, B′ helix and F′-G loop were quite limited to the specific zone (see Supplementary Video 3 movieMut1) represented by the cluster centroid and F helix, which experience large deformations in 1*; contrary Mut1 remains without deformation, which is in concordance with the data from ΔRMSF in which a rigid motion is found for this allelic form.

For Mut2, five clusters were found, and the representative structure for each cluster is illustrated by the structure found in the centroid (Fig. 4). Here, a characteristic motion was found for the F-F′ loop, F′ helix, B′ helix and F′-G loop, and a new deformation was observed at the end of the G helix; this could result from the mutation at position 253, where glutamate is changed to lysine, which is part of the G helix. A deformation was also found in the F helix, as observed for 1*. The deletion of Leu-241, which is part of the F′-G loop, was found in the allelic form 1*, where it participates in hydrophobic interactions with Ile-106 and Ile-109, restricting the motion between the F′-G loop and B′ helix. This can increase flexibility in the F-F′ loop, F′ helix, B′ helix and F′-G loop, which is related to the distance among them. Results of the cluster analysis showed extra conformations related to 1*, and this is in agreement with the data found in the ΔRMSF and PCA analysis (Figs 4 and S6 see Supplementary). Video 4 shows the 1000-sample structures (movie Mut2 see Supplementary).

To understand the changes in correlated motion between the mutants and 1*, a dynamic cross correlation analysis was performed using the Bio3D package^49^. Cross correlation between the i-th and j-th atoms is represented by the *C*_*ij*_ matrix, which ranges from −1 to +1. The *C*_*α*_ atoms for 1*, Mut1 and Mut2 were used to compute the cross correlation *C*_*ij*_ matrix. A positive value represents the correlated motion, and a negative value represents anti-correlated motion (see Supplementary Figs S8 and S9). We found that Mut1 presented more collective character than 1*. These strong correlations were observed between the B′ helix and F′-G loop, the B′ helix and helix G, the B′ and F′ and the F′ helix and *α* helix-*β*-1 loop. For Mut2, compared with 1*, strong correlations were also identified between the B′ helix and F′-G loop, the B′ helix and helix G, also with B′ and F′ and B′-G loop (see Supplementary Figs S8 and S9). Furthermore, the amount of anti-correlation motion for Mut2 increased significantly compared with that for 1*. These strong correlations found in Mut1 and Mut2 also support the different motions observed in the PCA analysis, where the porcupine graphics show different dynamics for the two allelic forms compared with 1* (see Supplementary Fig. S7). Contrary to the observed correlated motion, the anti-correlated motion is more evident in the case of Mut2 than for 1* or Mut1 (see Supplementary Figs S8 and S9), which is in agreement with the irregular flexibility observed for this allelic form and indicates some extra random motion, which is not concerted as for 1* and Mut1.

**Figure 5:**
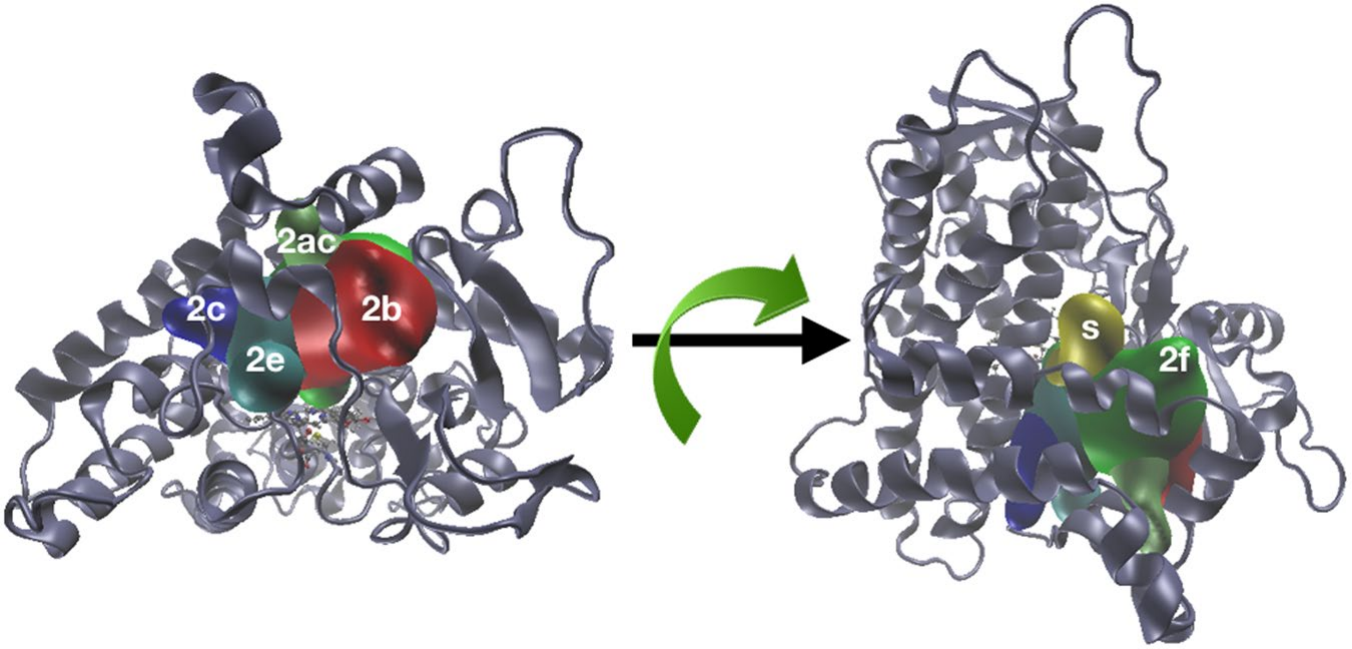
Principal tunnels identified in CYP2D6/1* throughout cluster analysis from MD simulations. Tunnels were determined from each cluster for CYP2D6/1*, Mut1 and Mut2 (only 1* is shown) The channels shown are 2c (blue), 2e (cyan), 2b (red), 2ac (lime), 2f (green) and Solvent (yellow).

Previously described for CYPs^27,50^, showing the principal access tunnels in the P450 2D6 protein, along with the dynamical behaviour for different allelic forms also in complex with ligands, describing dominant characteristics of the molecular tunnels^25,27^. Figure 5 shows the principal tunnels found for 1*. Tunnel calculation was performed for each set of structures that belong to the clusters for each allelic form (1*: four clusters, Mut1: one cluster, Mut2: five clusters). The bottleneck radius is shown in Fig. S10 (see Supplementary Fig. S10). For the three allelic forms, five classes of tunnels were identified (2b, s, 2e, 2f, 2c and 2ac) (Fig. 5). Tunnels 2b and 2e were found near to the B-B′ loop, with 2b opening on the side close to *β*-sheet and 2e in the middle of the B-C loop region. Tunnel 2c was found between the B-C loop and helix I. Tunnel s (solvent tunnel) was found between helices F and I; tunnel 2ac was located between the B′ helix and F′-G loop, and tunnel 2f was between F-F′ loop and A helix (Fig. 5).

In the case of 1*, for the clusters found in the cluster analysis, the bottleneck radius was plotted in each case (see Supplementary Fig. S10). For tunnel 2ac, cluster one shows two populations, with two characteristic peaks of approximately 1.02 and 1.38 Å (closed and opened tunnels), with the closed conformation being more common than the opened one in this cluster. For clusters two and three, a single population was found in both cases with a peak at 0.99 and 1.02 Å (closed conformation); for cluster four, opened and closed populations were found with characteristic peaks at 0.99 and 1.44 Å. Tunnel 2b, started in cluster one with a major population in a closed configuration, increasing the opened population in clusters four and two; whereas opened-tunnel population was found in cluster three. Closed tunnel 2c was found in cluster two, thus increasing the open populations in clusters one, three and four. The population in the closed conformation was still dominant, but an increase in the open population was also found. In the case of tunnel 2e, most of the population was in the closed conformation, causing a minor increase in the open conformation from clusters one, three and four. The solvent tunnel followed the same pattern as the previous tunnel description. An initial closed population was found in cluster two, increasing the amount of opened conformations starting in cluster three, followed by clusters one and four; thus, there were more open conformations than closed ones. In tunnel 2f, the closed conformation was evident in cluster one, indicating an increase in the open population in clusters three and four, in which almost 50% of the open population was found in cluster two. As described above, major structural changes in each cluster for the allelic form 1* were found in the F-F′ loop, F′ helix, B′ helix, F′-G loop and deformation of helix F (Fig. 4), exactly where tunnels are located in the enzyme. Furthermore, increased flexibility was found in the zone where the tunnels are located, as shown in the analysis of fluctuations (Figs 3 and S6 see Supplementary). The motion of the F-F′ loop, F′ helix, B′ helix, F′-G loop and helix F allows the dynamical behaviour of the tunnel, going from closed to open and vice-versa, depending of the conformation of these structural parts of the enzyme. Interestingly, there are no correlated motions in the secondary structures that form the tunnels, indicating that there is more than the ‘hatch’ proposed by Fukuyoshi and coworkers^22^ in which a concerted opened and closed motion is suggested. The entrance of the tunnel works like a ‘flexible door’, suggesting that a ligand with specific structural and physical properties can enter the tunnel and reach the active site without any concerted motion at the tunnel entrance.

For Mut1 (Fig. 4), we analysed the tunnels with the same parameters used for 1* (see Supplementary Fig. S10). We observed a difference in tunnel behaviour for Mut1 due to the bottleneck radius, which for all tunnels was found to be in a closed conformation (except for tunnel s, in which opened and closed populations were observed in that cluster). The Mut1 mutation produced rigid protein dynamics, as mentioned above for ΔRMSD, Fig. 3, and two new correlated interactions were found in the cross-correlation analysis, suggesting a concerted motion in the F′ helix, A-*β* 1 loop, B′ helix and F′-G (see Supplementary Fig. S8) as a consequence of the rigidity observed in the ΔRMSF. The above results indicate that to open the tunnels, it is necessary to cross a free-energy barrier because of the increased entropy in the protein that allows amino acids to move in an open conformation. As the experimental results have shown^2,18,20,21^, PMs cannot perform the enzymatic reaction. Our results for this allelic form suggest that the ligand cannot access the catalytic site because the Mut1 protein cannot move from the closed to open conformation (as the 1* protein can), at least in the first 400 ns of simulation.

In the case of tunnels for Mut2 (see Supplementary Fig. S10), we also observed different tunnel dynamics compared with the 1* protein. Contrary to Mut1, in Mut2, five clusters were found in the cluster analysis. Differences in dynamical behaviours were observed in Mut2 compared with 1* and Mut1. Tunnel 2c showed a larger open bottleneck radius than that in 1*. Cluster five showed a closed population, and cluster three showed an open population. However, clusters one and two were in intermediate states, and for cluster four, the tunnel was absent, indicating a closed conformation lower than the probe radius used for the analysis. Tunnel 2b showed an opened conformation in almost all the clusters, with tunnel number three having an open conformation in the majority of clusters. Tunnel 2c was found to be in the closed conformation, and tunnel 2c was only observed in clusters one and five. Tunnel 2e showed a closed conformation in clusters four, one and two but open conformation in cluster three. However, tunnel five was intermediate between opened and closed. Tunnel s showed both open and closed conformations for cluster four, an open conformation for cluster three and an intermediate conformation for clusters one, two and five. Finally, tunnel 2f showed a closed conformation for tunnel five, an open conformation for cluster two and an intermediate conformation for cluster one (see Supplementary Fig. S10). Open, intermediate (semi-open) and closed conformations were characteristic of Mut2 compared with 1*, and a larger open conformation was found for Mut2 (except for tunnel 2c, which had no open conformation).

The closed conformation of tunnel 2c was also common in Mut1, but in the case of Mut2, a deletion at 241-Leu significantly changed the dynamics of the protein. In the 1* protein, Leu-241 is localised at the beginning of the G helix and participates in hydrophobic interactions with amino acids of the B′ helix (Ile-106, Ile-109 and Leu-110). These interactions add space between the centre of mass of the amino acids Val-104 to Leu-110 (B′ helix), also with the beginning of G helix (Leu-241 to Phe2–247), of approximately 12 Å fluctuating from 10 to 13 Å, whereas for Mut2, the same measure shows two populations, the first one of approximately 10 Å and the second 12 Å (see Supplementary Fig. S11), with fluctuations from 9.4 to 13 Å. The dynamics of these two secondary structures showed a concerted motion in Mut2, and this was also observed in the cross-correlation analysis (see Supplementary Figs S8, S9 and S11). The effect of this phenomenon, produced by the deletion of Leu-241, was keeping the tunnel 2c closed. Another effect of this deletion could be to modify how tunnel 2ac recognises the ligand and to modify the shape of tunnel 2b. This may be one reason for the higher bottleneck radius of tunnel 2b.

## Discussion

In this study, we found that in the PM group, the average endoxifen concentration was approximately 2.57 ng/mL. This is similar to results in previous studies^21,34–36^ in which the highest endoxifen concentrations were identified and correlated with different allelic forms of CYP2D6 in patients with infiltrating ductal carcinoma. However, endoxifen concentrations were varied, possibly indicating that the genotype affected the endoxifen concentration. Nonetheless, data for the IMs showed a mean deviation towards lower endoxifen values than expected values. Here, the limitation of the data should be taken into account; patients may not have adhered to their treatment regimen, possibly affecting the plasma endoxifen concentration. In addition, patients may have used other drugs metabolised by CYP2D6, and this should be taken into consideration^38,39^.

The CYP2D6 phenotypes are stratified into well-defined categories. These are: PM, IM, EM and UM. Here, we identified the CYP2D6*9 allelic variant in a Colombian population for the first time (CYP2D6*9) and characterised the PM *4/*41 and *4/*9 forms. These two PM patients were of interest because they have the risk of disease relapse, as changing the dose of tamoxifen will likely be ineffective. Molecular simulations allow an atomic-level description of the possible effects of specific mutations. Thus, we performed molecular dynamics analyses on CYP2D6 to understand the dynamics of tunnel formation and ligand interactions. We conduct this study using all possible tunnel that can form for the CYP2D6, in which tamoxifen can access to the catalytic site and this because to date, there is not experimental evidence about the exact site where tamoxifen can entrance. However, studding all possible access sites, can offer an approximation about the relationship between a specific mutation and the dynamical behavior of the protein. Thus, one can extrapolate what happens when tamoxifen try to access at the catalytic site, if the tunnel is affected in some extend. For Mut1, the majority of tunnel entrances accessible by tamoxifen remain closed in 400 ns of simulation. As a consequence, compared with 1*, tamoxifen has a small probability to enter the active site. Mut1 affects the plasticity of the protein, making it more rigid.

In the case of Mut2, the effect of the mutations are completely different from CYP2D6*1 and Mut1. Mut2 is highly flexible; the correlated and anti-correlated motion of the tunnel bottleneck changed from CYP2D6*1, completely altering the dynamics of the protein and affecting the entrance of tamoxifen into the active site. These results are in agreement with the fact that PM patients with these allelic forms cannot metabolise tamoxifen to endoxifen. These mutations make CYP2D6 inefficient, with serious implications for a patient treated with tamoxifen; specifically, treatment will be ineffective, increasing the risk of developing BC or relapsing. This is the first study in Colombia to report the close relationship between different allelic forms of CYP2D6 and the metabolism of tamoxifen in patients with BC. Our results show the relevance of conducting pharmacogenetics studies of patients receiving the same treatment but who display a different metabolic response. Because of the diversity of allelic forms in a population, it is very important to implement population genetics studies through the country’s health system to collect information and develop a personalised treatment for each patient.

## Methods

### Population

Thirty patients diagnosed with infiltrating ductal carcinoma who were treated with tamoxifen were selected from the San Ignacio University Hospital (HUSI). This study was approved by the Universidad National de Colombia Ethics Committee (10 June 2014) and the HUSI Ethics Committee (FM-ICD-7038-13). Written informed consent was obtained from all patients before participation in the study. Authors confirm that all research was performed in accordance with regulations of the local ethics committee.

### Sample handling and DNA extraction

Blood (4 mL) was collected at a baseline clinic visit, placed on ice, and within the hour separated into plasma, buffy coat and red blood cells using centrifugation at 2300 x g at 4 °C for 10 minutes. All samples were immediately frozen. Aliquots were stored at −80 °C in cryogenic tubes until analysis. Genomic DNA was extracted from these archival samples using 200 *μ*L of the buffy coat fraction (“UltraClean Blood DNA Isolation Kit (Non-Spin)” Mo Bio) was quantified with a Nanodrop 1000 spectrophotometer (Thermo Fisher Scientific, Waltham, MA, according to manufacturers’ instructions).

### CYP2D6 Genotype and Phenotype

Genomic DNA was isolated from whole blood collected at enrolment and used for CYP2D6 genotyping using the QuantStudio 12k Flex Real-Time PCR System with TaqMan SNP Genotyping Assays on OpenArray Plates and TaqMan Copy Number Assays. The QuantStudio platform can identify 23 distinct alleles and known duplications, which were then combined to predict a CYP2D6 phenotype (PM, IM, EM or UM)^51^.

### Tamoxifen and Endoxifen Concentrations

The plasma concentrations of tamoxifen, (Z)-4-hydroxy-tamoxifen, N-desmethyl-tamoxifen and endoxifen were measured in the blood samples collected at enrolment (at which point all patients had been on tamoxifen at a dose of 20 mg/day for at least 4 months) using a high-performance liquid chromatography/tandem mass spectrometry (API 3200) assay^52^.

### Computational methods

From the 2.8 Å resolution X-ray structure of CYP2D6 bound to prinomastat (PDB ID: 3QM4)^53^, the protein of interest was constructed as an initial model for running molecular dynamics (MD). Chain A and its corresponding heme cofactor were used, whereas prinomastat and crystallographic water were removed. Mutations Mut1: Ser-488-Thr/Arg-329-Cys/Pro-487-Ser and Mut2: Ser-488-Thr/Glu-253-Lys/Pro-487-Ser/Deletion-Leu-241 were simulated from the minimised initial structure using backbone-dependent rotamers from the UCSF chimera package^54^. Minimisation and MD protocols were performed with AMBER 14^55^. The force field parameters for amino acid residues were ff14SB43^56^. For heme cofactor, a high spin penta-coordinated from quantum mechanically derived and Giammona parameters were used^57^. The 1*, Mut1 and Mut2 structures were simulated to all-atom, unrestrained molecular dynamics simulations in explicit solvent using the GPU version PMEMD engine provided with Amber14. The Leap module integrated with Amber14 was used to add missing hydrogen atoms and Na^+^ counter ions for neutralisation. The three systems were immersed into an orthorhombic box using the water model TIP3P^58^. The long-range electrostatic interactions were computed using the particle mesh Ewalds method^59^, with a direct space and vdW cut-off of 12 Å. An initial minimisation using a retrained potential of 500 kcal mol^−1^ Å^2^ was applied to the solute for 1000 steps of the steepest descent algorithm, followed by 1000 steps of the conjugate gradient method. Subsequently, 1000 minimisation steps were simulated without any restraints using a conjugated gradient algorithm. The heating protocol was performed with a gradual temperature increase from 0 to 310 K using harmonic restraint of 5 kcal mol^−1^ Å^2^, which was applied to the solute; a Langevine thermostat with a collision frequency of 1 ps^−1^ was used with the canonical ensemble (NVT). The three systems were equilibrated at 310 K in an NPT ensemble for 5 ns without any restraint and using Berendsen barostat to maintain the pressure at 1 bar. The SHAKE algorithm^60^ was used to constrain the bonds of all hydrogen atoms, and a time step of 2 fs and the SPFP precision model^61^ were used in all MD simulations. Finally, 400 ns of production were simulated for each allelic form in an NPT ensemble with a target pressure of 1 bar and a pressure coupling constant of 2 ps. The production trajectories were analysed for 2 ps of simulation using CPPTRAJ and PTRAJ. RMSD and RMSF were computed, and PCA was performed for the backbone atoms (*Cα*, C and N). PCA was performed for Ca atoms of the three allelic forms, previous water and ion removal and alignment using as a reference the minimised structure. PCA was initially performed for the complete set of coordinate data, and the first two principal components, which correspond to the first two eigenvectors of the covariance matrix, were analysed. Subsequently, a sample of 1000 coordinates from the total data were taken, and a PCA analysis was performed over the new set of coordinates. Cluster analysis (based on k-means) of the new data set was performed with the MMTSB tool set^62^, with a fixed cluster radius of 1.7 Å for the three systems, taking into account only the coordinates of the *Cα* atoms, and subsequently, the clusters obtained were projected onto the two principal components. Finally, tunnel analysis was performed for the structures in each cluster, for a total of 1000 coordinates per system. The CAVER 3.0 package was used for the access and egress tunnels^50^. The starting point was set to 4.0 Å above the iron atom in the cytochrome cofactor. The clustering threshold was set up to 4.0 Å and the probe radius to 0.9 Å; standard settings for CAVER 3.0 were used for the remaining clustering settings.

## Supplementary information

Video 1

Video 2

Video 3

Video 4

Supplementary information

T1

T2

## Data Availability

Files used for molecular dynamics simulation can be found in https://github.com/jyosa/CYP2D6.
