# Supplementary material for: Dynamic Effects of CYP2D6 Genetic Variants in a Set of Poor Metaboliser Patients with Infiltrating Ductal Cancer Under Treatment with Tamoxifen
Source: Sci Rep. 2019 Feb 21;9:2521. doi: 10.1038/s41598-018-38340-6 (PMC6385267; doi:10.1038/s41598-018-38340-6)
Supplement: Supplementary file 5 — Supplementary information [file 41598_2018_38340_MOESM5_ESM.pdf]

Supplementary Information

Dynamic Effects of CYP2D6 Genetic Variants in a Set of Poor Metaboliser Patients with Infiltrating Ductal Cancer Under Treatment with Tamoxifen

Yeimy Viviana Ariza[1,+], Ignacio Briceño[2,3], Fabio Aristizábal[1], Luis Fernando Niño[4], and Juvenal Yosa Reyes[5,+,*]

[1]Universidad Nacional de Colombia, Biotechnology Institute, Bogota, 111321, Colombia

[2]Pontificia Universidad Javeriana, Institute of Human Genetics, Bogota, 11001000, Colombia

[3]Universidad de la Sabana, Department of Pharmacy, Bogota, 140013, Colombia

[4]Universidad Nacional de Colombia, Department of Computer Science, Bogota, 111321, Colombia 5, Universidad Simón Bolivar, Molecular Simulation and Bioinformatics Lab, Barranquilla, 080002, Colombia * juvenal.yosa@unisimonbolivar.edu.co

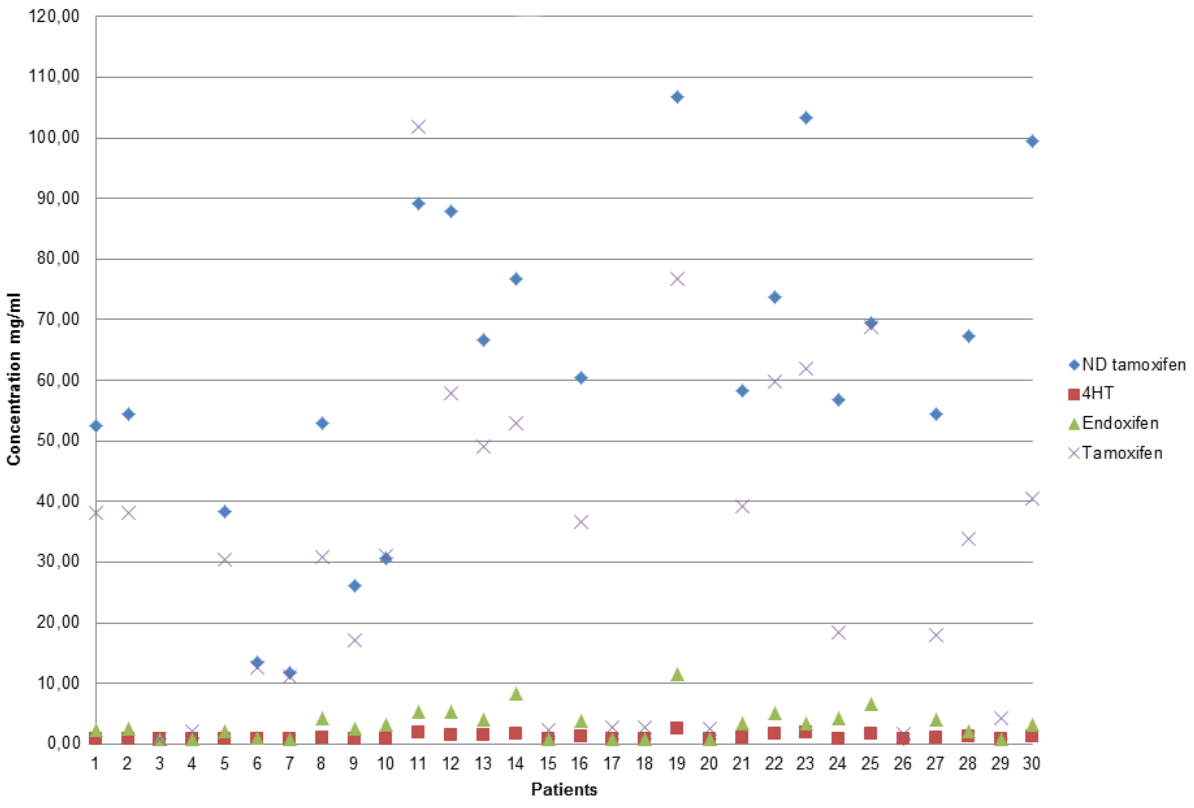

Figure S1. Concentration of ND-Tamoxifen (blue diamonds), 4HT = 4 hydroxy-tamoxifen (green) and tamoxifen (gray). On the X-axis each of the 30 patients is represented and on the Y-axis, the concentrations are given in mg/mL.

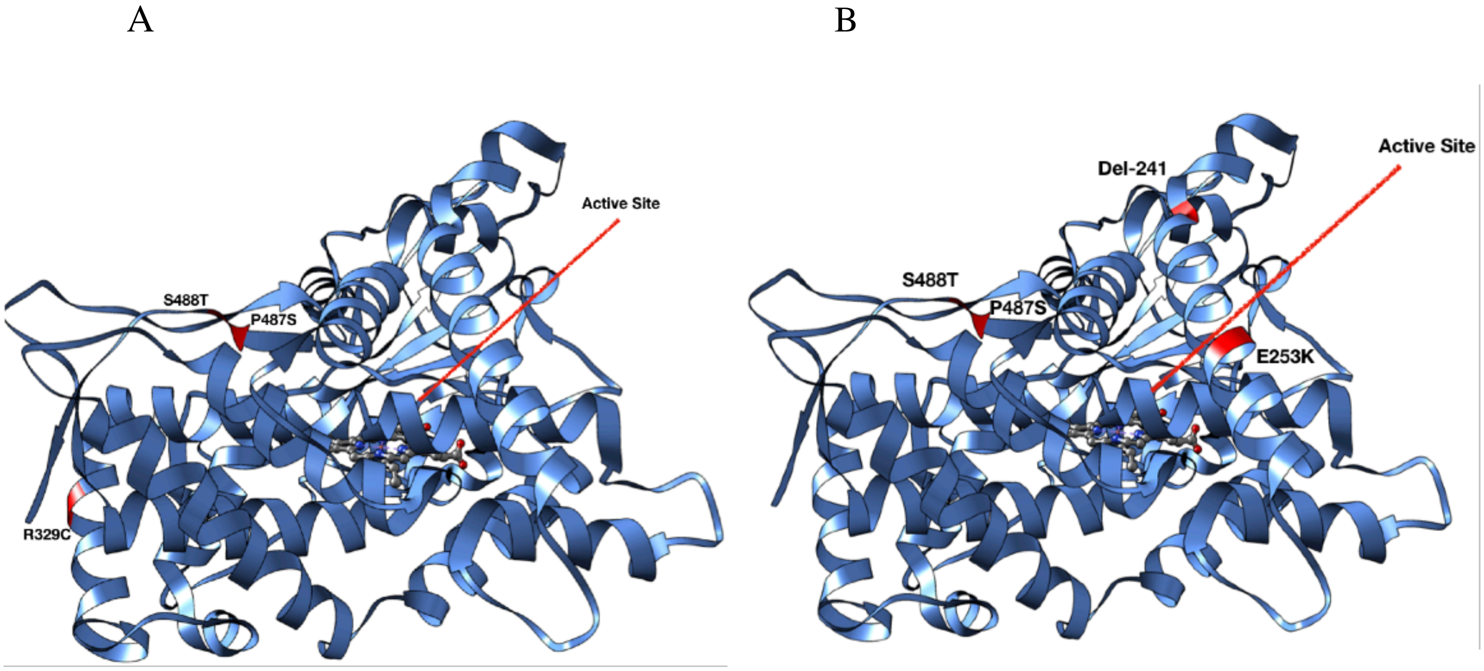

Figure S2. CYP2D6 SNPs in the two PM patients. A) patient 1 Ser-488-Thr/Arg-329-Cys/Pro-487-Ser (termed Mut1), B) patient 2 Ser-488-Thr/Glu-253-Lys/Pro-487-Ser/Deletion-Leu-241 (termed Mut2). SNPs are located distal from the active site.

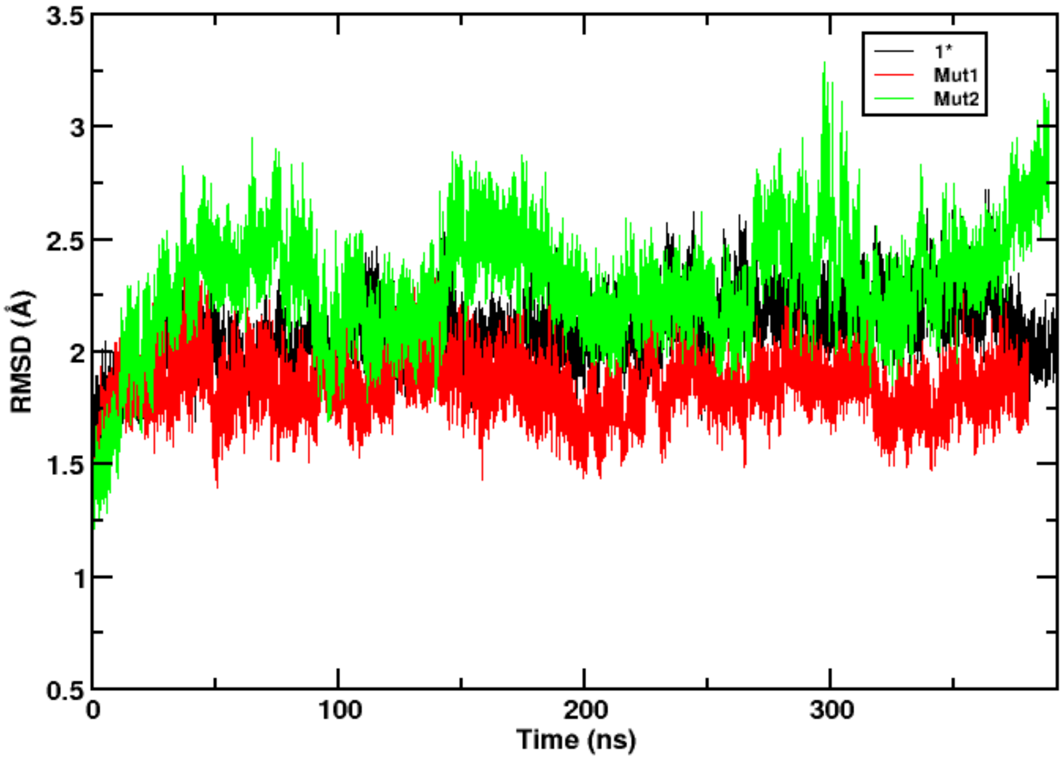

Figure S3. Root mean square deviations (RMSDs) for 1*, Mut1, and Mut2 of CYP2D6.

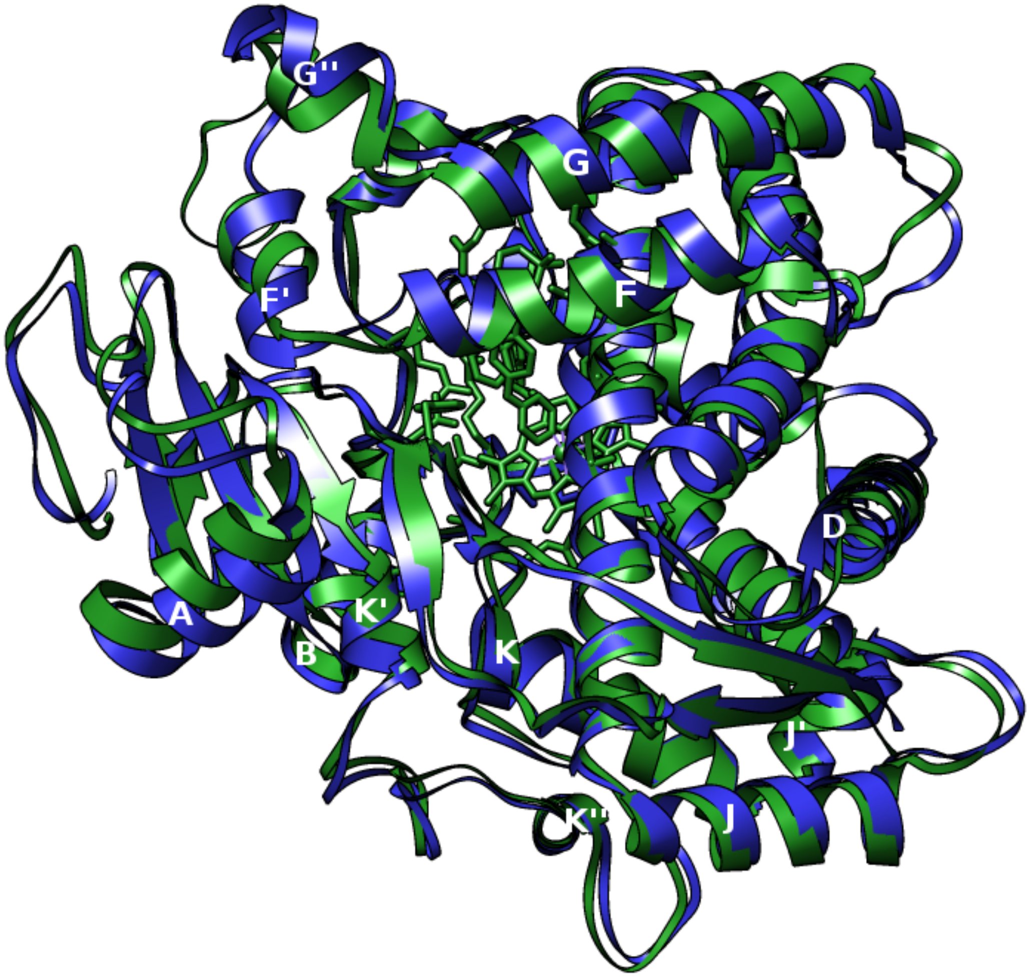

Figure S4. Superposition of CYP2D6/1* (blue) and experimental structure 3QM4 (green). computed RMSD 1.113 Å.

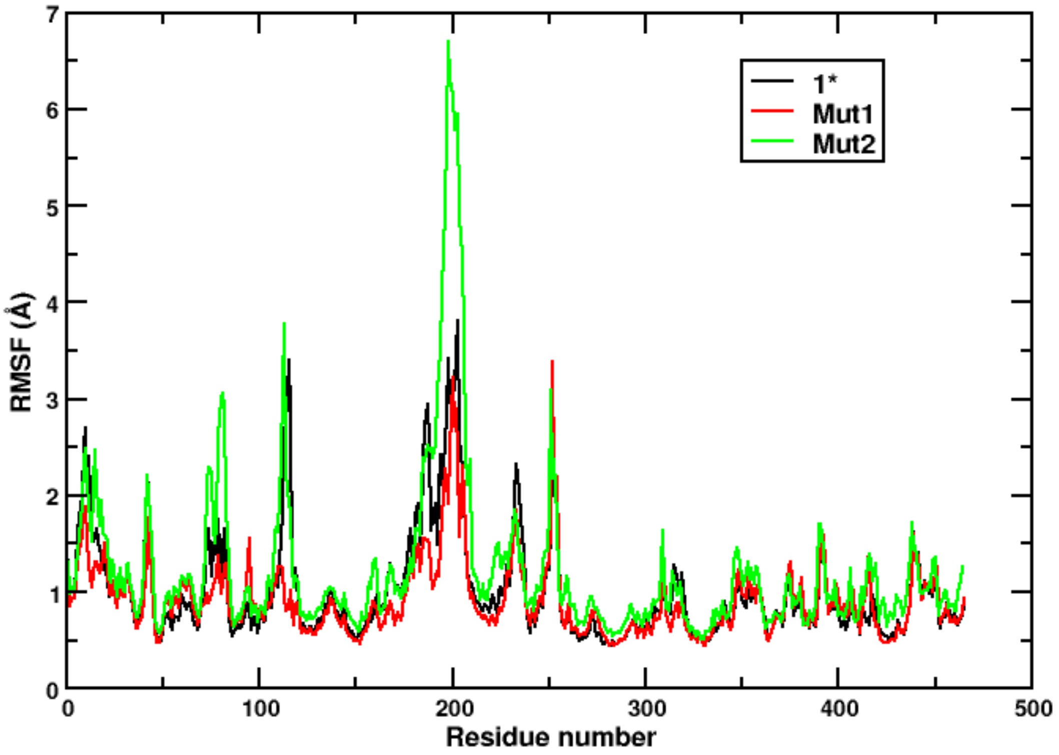

Figure S5. The-root-mean square fluctuation (RMSF) of backbone atoms; the values were calculated from 50 to 400-ns of molecular dynamics trajectories.

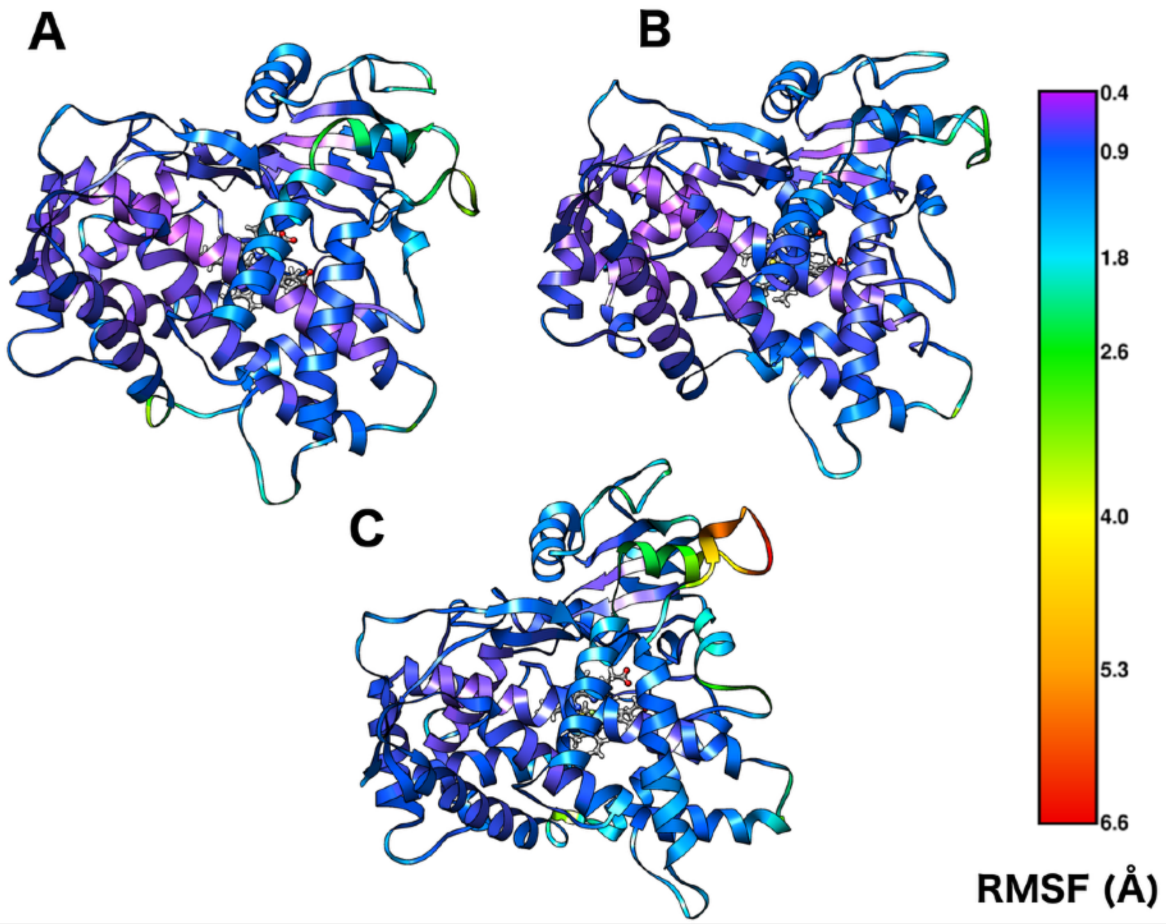

Figure S6. Structural fluctuations of each CYP2D6 A) 1*, B) Mut1 and C) Mut2. The color scheme indicates the degree of fluctuation, going from violet indicating little fluctuation to red indicating large fluctuation.

A

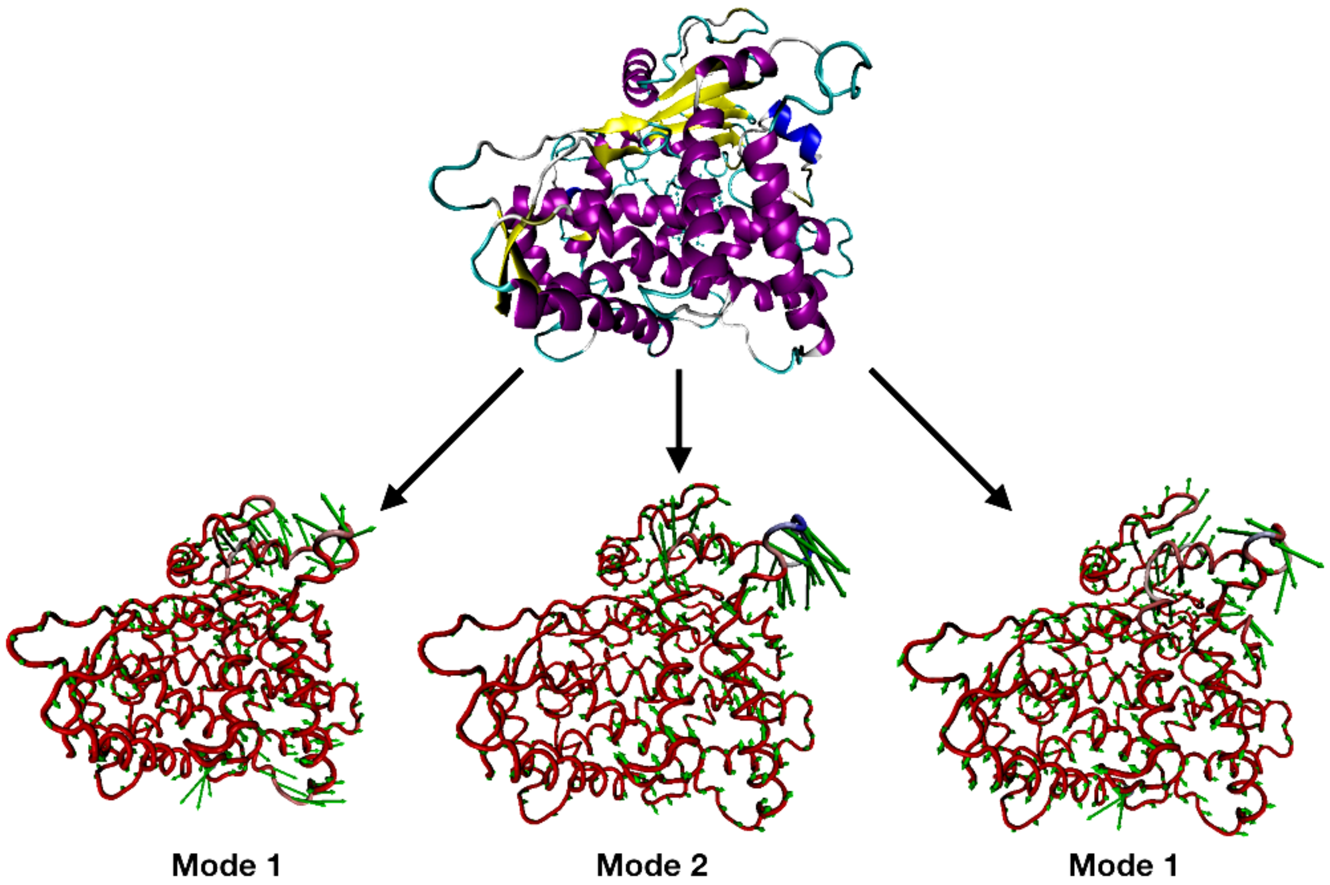

B

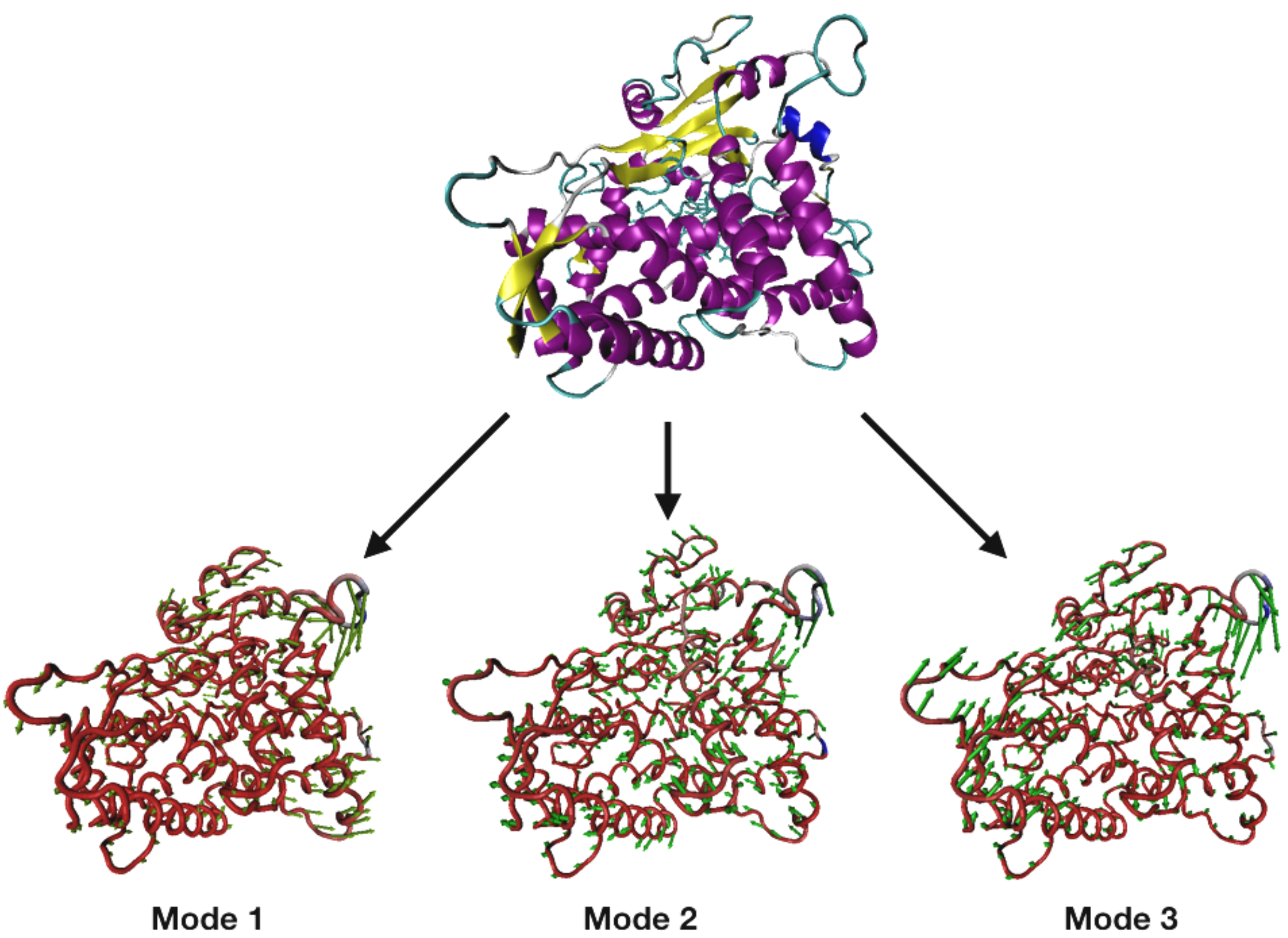

C

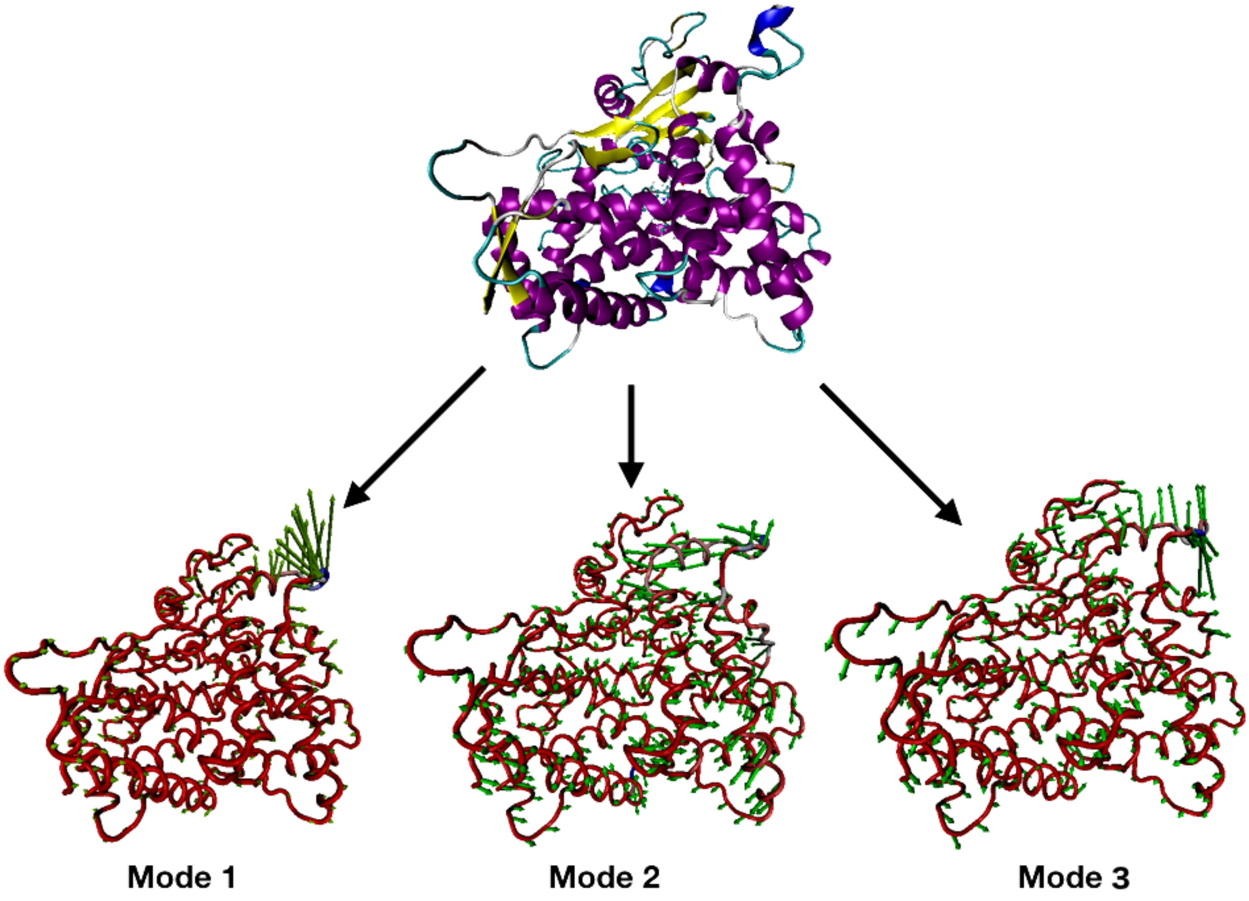

Figure S7. Porcupine plot analysis. The porcupine plot of the first three eigenvectors of (a) 1*, (b) Mut2 and (c) Mut2 allelic forms. The arrows indicate the direction of the eigenvector and magnitude of the corresponding value.

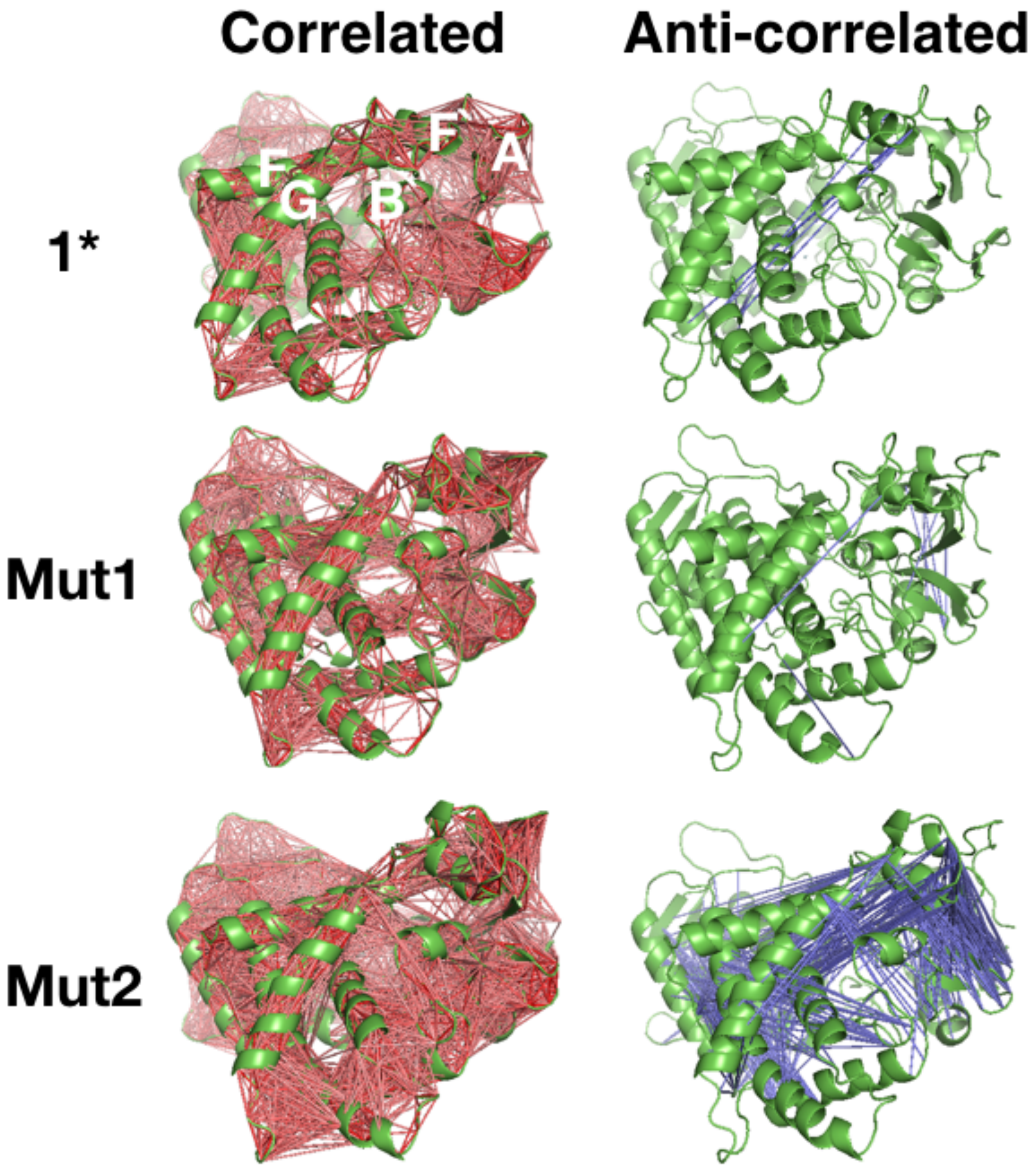

Figure S8. Cross-correlation matrix of Cα atoms during 350 ns of simulation for A) 1* type, B) Mut1 and type C) Mut2. The motion is indicated in various colors on the panel. Aquamarine indicates a positive correlation, whereas magenta indicates anti-correlation.

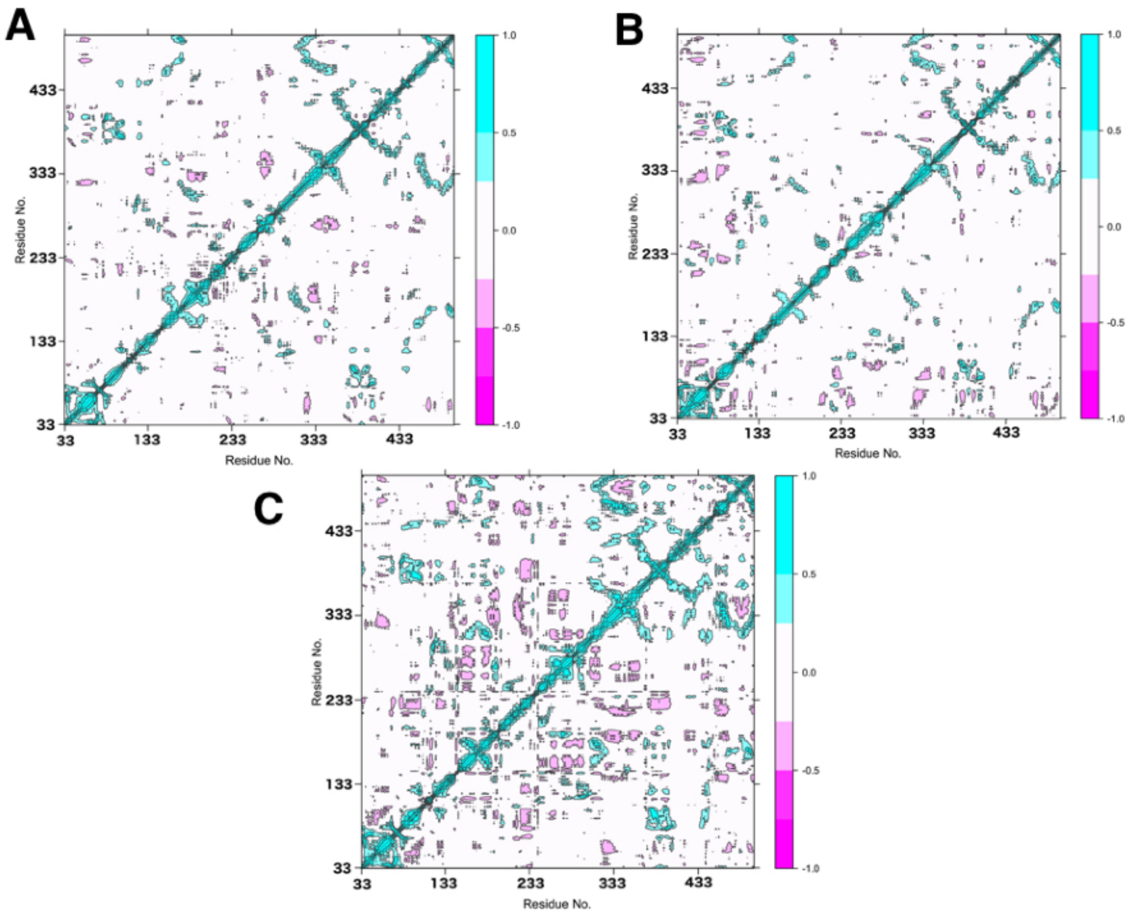

Figure S9. Residue-residue cross-correlations. Visualization of residue-residue cross-correlations. Red and blue lines indicate correlated and anti-correlation motions, respectively. Mut1 presents more collective character compared with 1*. These strong correlations are observed between B′helix and F′-G loop, B′ helix and helix G, B′ and F′, F′ helix and A helix-β1 loop, compared with 1*. A strong correlation was also identified for Mut2 between B′ helix and F′-G loop, B′ helix and helix G, B′ and F′ and B′-G loop.

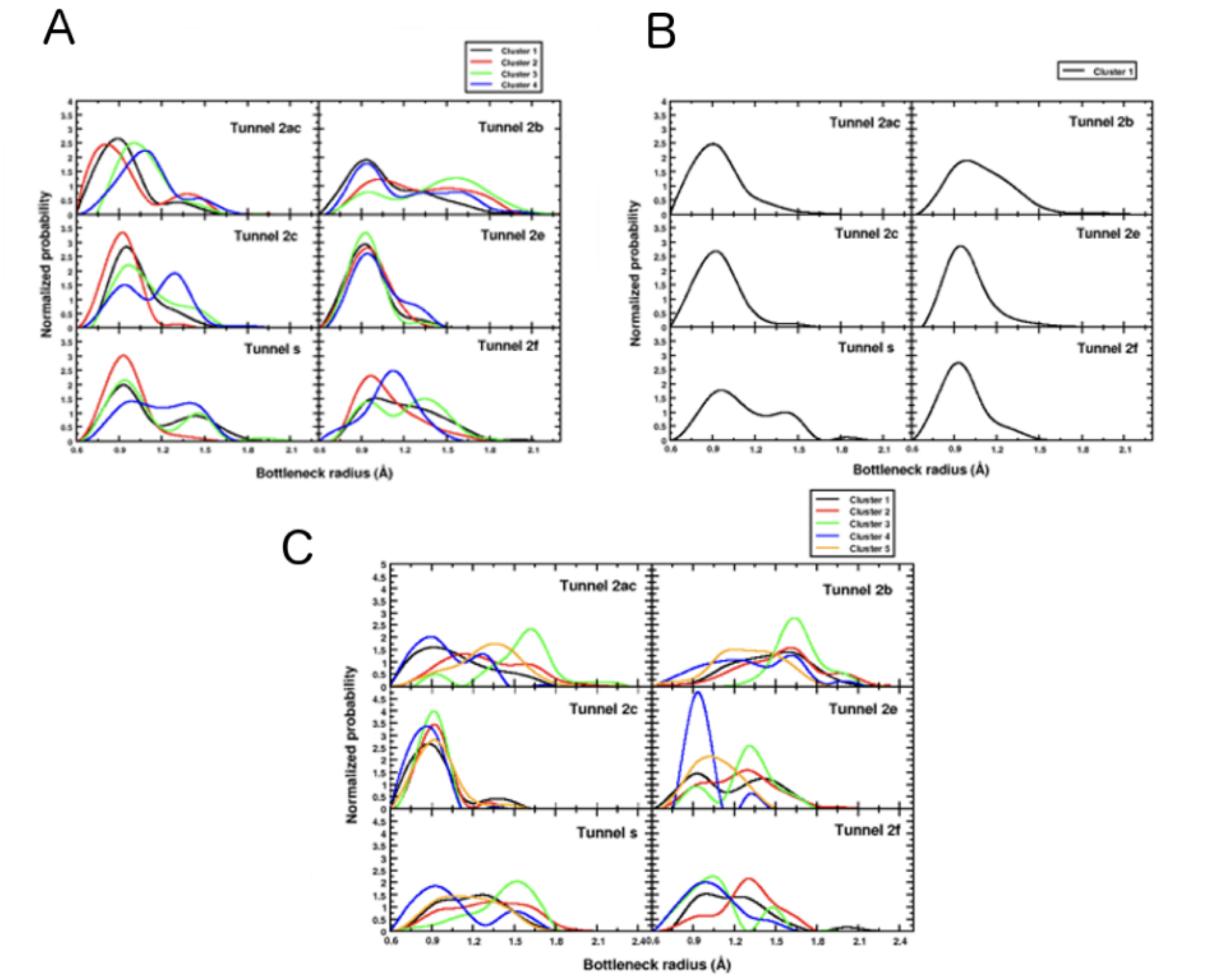

Figure S10. Bottleneck radius distribution for (A) CYP2D6/1*, (B) Mut1 and (C) Mut2. distributions are plotted according to the clusters obtained using k-means. Colors are labeled according to the same pattern as in Figure Figure 4, Black for cluster one, Red for cluster two, Green for cluster three, Blue for cluster four and Orange for cluster five.

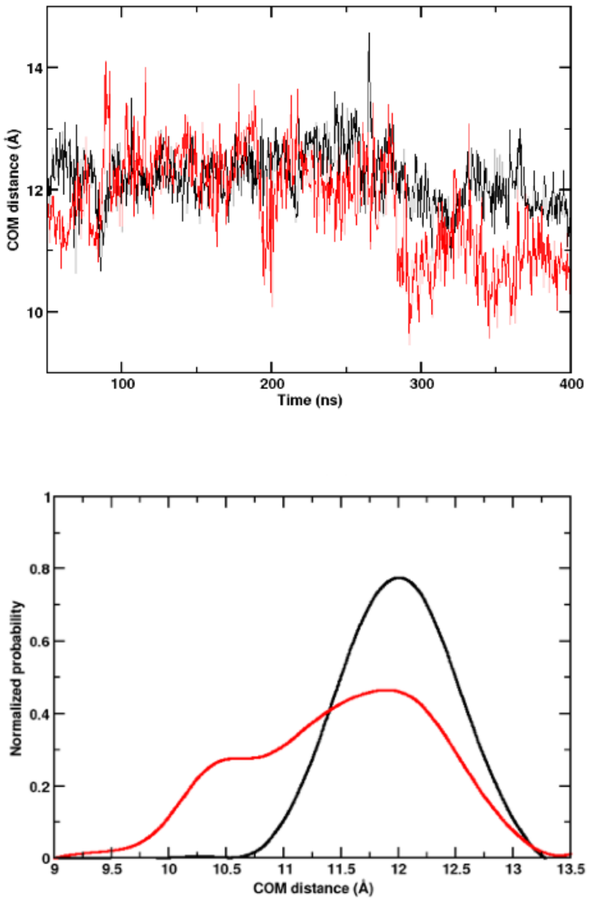

Figure S11. The distance between the center of mass of the amino acids Val-104 to Leu-110 (B′ helix) and the beginning of G helix (Leu-241 to Phe-247) and distributions of distance in 350 ns simulations. Black color corresponds to 1* and red color to Mut2.
